# Norovirus VPg Binds RNA through a Conserved N-Terminal K/R Basic Patch

**DOI:** 10.3390/v13071282

**Published:** 2021-06-30

**Authors:** Alice M. McSweeney, Vivienne L. Young, Vernon K. Ward

**Affiliations:** Department of Microbiology & Immunology, School of Biomedical Sciences, University of Otago, P.O. Box 56, Dunedin 9054, New Zealand; alice.mcsweeney@otago.ac.nz (A.M.M.); vivienne.young@otago.ac.nz (V.L.Y.)

**Keywords:** norovirus, calicivirus, RNA binding, VPg, spidroin

## Abstract

The viral protein genome-linked (VPg) of noroviruses is a multi-functional protein that participates in essential roles during the viral replication cycle. Predictive analyses indicate that murine norovirus (MNV) VPg contains a disordered N-terminal region with RNA binding potential. VPg proteins were expressed with an N-terminal spidroin fusion protein in insect cells and the interaction with RNA investigated by electrophoretic mobility shift assays (EMSA) against a series of RNA probes (pentaprobes) representing all possible five nucleotide combinations. MNV VPg and human norovirus (HuNV) VPg proteins were directly bound to RNA in a non-specific manner. To identify amino acids involved in binding to RNA, all basic (K/R) residues in the first 12 amino acids of MNV VPg were mutated to alanine. Removal of the K/R amino acids eliminated RNA binding and is consistent with a K/R basic patch RNA binding motif within the disordered N-terminal region of norovirus VPgs. Finally, we show that mutation of the K/R basic patch required for RNA binding eliminates the ability of MNV VPg to induce a G0/G1 cell cycle arrest.

## 1. Introduction

Human noroviruses (HuNV) are a significant cause of gastroenteritis and account for approximately one-fifth of acute gastroenteritis across all age groups [1]. Recent studies have demonstrated HuNV replication in a continuous human B cell line, stem cell derived human enteroids or zebrafish larvae, significantly increasing our ability to directly study HuNV [2,3,4]. However, implementation of these systems within the laboratory can be complex and there is considerable variation in replication between HuNV strains [5]. As a result, murine norovirus (MNV) remains a commonly utilised model virus for studying the viral lifecycle.

MNV replicates in laboratory cell culture lines and retains a similar genomic layout to HuNV [6,7]. MNV possesses a positive-sense single-stranded RNA genome with four open reading frames (ORF). ORF1 is translated as a large polyprotein, which is cleaved by the viral protease into the six non-structural proteins, NS1-2, NS3, NS4, NS5 (VPg), NS6 (Pro), and NS7 (Pol, RdRp) [8]. ORF2 and ORF3 encode the major and minor capsid proteins, respectively, while the fourth ORF of MNV encodes a virulence factor [9].

**Figure 1:**
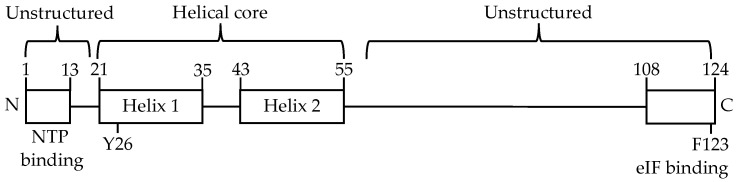
Schematic of MNV VPg. The structural helices and helical core are indicated. Numbers represent the relative positions of amino acids. Amino acid Y26 is essential for nucleotidylylation and F123 is essential for translation. The unstructured N-terminal and C-terminal ends of the protein are indicated. The first 13 amino acids have been shown to be involved in NTP binding by the VPg of HuNV.

As with many viral proteins, the VPg proteins of noroviruses are multi-functional with a range of activities critical to the lifecycle of the virus. Nuclear magnetic resonance (NMR) analysis has shown that MNV VPg consists of a structured helical core flanked by unstructured regions at each end of the protein (Figure 1) [10]. During replication, VPg acts as protein primer through the covalent addition of a nucleotide to a conserved tyrosine residue (Y26 in MNV) which then functions as the first base for extension by the RNA polymerase during genome replication [11,12,13]. VPg is required for recruitment of the host eukaryotic initiation factor 4G, facilitating translation of the viral genome [14,15,16]. VPg, and in particular the N-terminal region of the protein, is also important for induction of a G0/G1 phase cell cycle arrest shown to promote viral replication [17,18,19].

VPg proteins are not unique to caliciviruses and have been identified in viruses in the *Picornaviridae* and *Potyviridae* families, among others. The VPg proteins in these families share some similarities, including nucleotidylylation, however, additional functions and sequences of the proteins vary [20,21]. Non-calicivirus VPg proteins, either as mature or precursor proteins, have been shown to bind RNA [22,23,24] but binding of calicivirus VPg proteins to RNA is unknown. Despite an absence of canonical RNA binding motifs, MNV VPg has been shown to bind phosphocellulose [20], a characteristic of RNA binding proteins. The N-terminal region of HuNV VPg has also been shown to bind nucleotide triphosphates (NTPs) [25]. Recently, it has become apparent that the traditional view of canonical RNA binding motifs is too narrow, and disordered regions of proteins represent a significant proportion of RNA binding proteins [26,27,28].

We identified a region of MNV VPg within the disordered N-terminus as a putative RNA binding site. RNA electromobility shift assays (EMSA) showed that MNV VPg and HuNV VPg were able to directly bind to RNA in a non-specific manner and we show that the MNV VPg binds to RNA through a basic patch of charged amino acids within the disordered N-terminal region that is conserved in noroviruses. Finally, we show that perturbation of the N-terminal amino acids critical for RNA binding also affects the ability of VPg to induce a cell cycle arrest.

## 2. Materials and Methods

VPg protein sequences for MNV and HuNV GII.4 strain Sydney 2012 were obtained from GenBank (accession numbers DQ285629 and JX459908, respectively). The nucleic acid binding potential of MNV VPg was assessed using the DRNAPred server [29]. Protein sequences were submitted to PrDOS [30], PONDR VL-XT [31,32,33] and IUPred2A [34,35] servers for prediction of disorder in the first 35 amino acids of MNV VPg using the default settings.

### 2.1. Expression and Purification of Wild-Type and Mutant VPg Proteins

Synthetic genes for MNV VPg, including a K5A/K7A/R10A triple mutant (TM), a mutant with all K and R residues in the first 12 amino acids of VPg modified to alanine (K2-12), HuNV GII.4 VPg and *E. coli* chloramphenicol acetyltransferase (CAT) (GenBank BBB37551) were synthesised by Genscript (Piscataway, NJ, USA). All constructs were confirmed by sequencing. Genes were ligated into a baculovirus transfer vector after a p10 promoter and recombinant baculovirus was produced using the Flashback Ultra system (Oxford Expression Technologies, Oxford, UK). A solubility tag, spidroin NT* [36,37], was engineered as a fusion protein to the N-terminus of VPg, which contained a N-terminal His_6_-Strep II tandem affinity tag, the spidroin NT* fusion partner, an enterokinase cleavage site, and a flexible GGSRS linker, followed by the VPg or CAT protein.

For protein expression, suspension cultures of *Trichoplusia ni* cells were infected with recombinant baculovirus expressing VPg proteins or CAT at a multiplicity of infection of one and incubated at 27 °C at 125 RPM for three days. Cells were harvested by centrifugation and lysed in 50 mM Tris pH 8.0, 150 mM NaCl, 10% glycerol, and 1% triton X-100. The protein was purified on Streptactin XT superflow beads (IBA Lifesciences, Gottingen, Germany) and eluted with 50 mM Tris pH 8, 150 mM NaCl, 10% glycerol, and 50 mM Biotin. Protein concentration was determined by absorbance at 280 nm on a NanoDrop One (Thermo Fisher Scientific, Waltham, MA, USA) and eluted proteins were stored at −80 °C. Expression and purification of recombinant proteins was confirmed by SDS-PAGE gel with Coomassie blue G250 staining.

### 2.2. Generation of RNA Probes and mRNA Transcripts

The RNA binding potential of VPg was screened against a series of plasmids containing 12 pentaprobes (PP) gifted by Professor Joel Mackay (Addgene plasmid # 83326–83337) [38]. Purified plasmid DNA was linearised with ApaI and single-stranded RNA (ssRNA) was transcribed for each pentaprobe sequence under control of a T7 promoter with incorporation of fluorescent rUTP ATTO 680 (Jena Biosciences, Jena, Germany) for detection. The transcription reaction consisted of 4 μL of 5× Transcription Optimised buffer (Promega Corporation, Madison, WI, USA), 1 μg of linear DNA template, 0.5 mM rATP, 0.5 mM rCTP, 0.5 mM rGTP, 12.5 μM rUTP (Promega), 20 μM rUTP ATTO 680, and 40 U T7 RNA polymerase (Promega), in a final reaction volume of 20 μL with nuclease free water. The transcription reaction was incubated in the dark at 37 °C for two hours. The DNA was degraded with DNase (Promega) and the RNA purified using a MEGAclear^TM^ Transcription Clean-Up kit according to the manufacturer’s instructions (Ambion, Austin, TX, USA). Labelled RNA probes were eluted in nuclease free water and stored at −80 °C.

RNA probes, approximately 150–200 bp, were designed to represent the 5′ and 3′ ends of the positive and negative-sense MNV RNA. The constructs were designed to include prominent RNA secondary structure elements near the 5′ and 3′ termini of the genomic and anti-genomic RNA. DNA templates for RNA probes were isolated by PCR and cloned into a pUC57 vector. All primers included a T7 promoter sequence and EcoRV restriction enzyme site, in addition to EcoRI and BamHI sites for cloning of the PCR products into the pUC57 plasmid (Table 1).

The 5′pos probe corresponding to the 5′ end of the positive-sense RNA represents nucleotides 1–155 of the MNV-1 genome and includes the 5′UTR and three stem loop RNA structures present at the 5′ end of the ORF1 sequence. The 3′neg probe corresponds to the 3′ end of the negative-sense RNA and is the mirror of the 5′pos probe. The 3′pos probe corresponds to the 3′ end of the positive-sense RNA, representing nucleotides 7158–7392 of the MNV-1 genome. The 3′pos probe contains three prominent stem-loops, the 3′ UTR and a short poly(A) tail. The 5′neg probe corresponding to the 5′ end of the negative-sense RNA is the complement of the 3′pos. The recombinant plasmids carrying the terminal sequences were linearised with EcoRV and RNA produced as described for the pentaprobes.

Generation of messenger RNA transcripts for transfection of RAW-Blue cells was performed as described previously [18]. Briefly, plasmids were linearised at the 5′ end using EcoRI and mRNA transcripts were produced using the mMessage mMachine T7 ultra transcription kit (Ambion). RNA was purified using the MEGAclear^TM^ Transcription Clean-Up kit according to the manufacturer’s instructions (Ambion).

Unlabelled DNA for competition assays was either produced from a single-stranded DNA template (TATGCGGGCGAAACTTCTGGGAATAGTCCTGACAACCCCTATTGCGATCAGCTCTTTTGCTAGCTCG) or a complementary sequence was annealed to the single-stranded DNA to produce double-stranded DNA.

### 2.3. RNA Binding Assays

Purified VPg proteins were buffer exchanged into RNA binding buffer (10 mM MOPS pH 7.0, 50 mM KCl, 5 mM MgCl_2_ and 10% glycerol). Pentaprobe RNA was heated to 95 °C for one minute and then immediately cooled on ice prior to use, viral RNA probes were not heat denatured prior to the addition of VPg. For RNA binding reactions, ~100 nM of ATTO680 labelled RNA was added to protein samples at the indicated concentrations. The reaction was incubated in RNA binding buffer at 4 °C for 30 min. For DNA competition assays, a 100-fold molar excess of unlabelled DNA probe was also added to the binding reaction. Binding reactions were loaded onto a 5% native TBE acrylamide gel (29:1 acrylamide: bis-acrylamide) and electrophoresed in 0.5× TBE buffer at 120 V for approximately 40 min at room temperature. Gels were imaged on an Odyssey Fc imager in the 700 nm channel.

### 2.4. Cell Cycle Analysis

Cell cycle analysis was performed as described previously [18,19]. Briefly, 1 × 10^6^ RAW-Blue cells were transfected with 4–5 μg of in vitro transcript mRNA using an Invitrogen Neon transfection system (Thermo Fisher Scientific). Cells were incubated for 12 h and harvested in 70% ethanol, washed in PBS (Dulbecco’s A), the RNA degraded with 0.1 mg/mL RNase A, and DNA stained with 50 μg/mL propidium iodide. The percentage of cells in each phase of the cell cycle was measured by fluorescence-activated cell sorting and the phases of the cell cycle assigned using MODfit LT 5.0 software (Verity Software House; Topsham, ME, USA). Expression of VPg proteins was confirmed by western blot using the appropriate primary and secondary antibodies; polyclonal rabbit anti-MNV-1 VPg [39], goat anti-actin (I-19) (sc1616; Santa Cruz, CA, United States), DyLight 800 donkey anti-rabbit IgG (SA5-10050; Thermo Fisher Scientific), and DyLight 680 donkey anti-goat IgG (SA5-10090; Thermo Fisher Scientific).

Cell cycle data presents the mean and standard deviation of three independent experiments. Results were analysed by one-way ANOVA with a Dunnett’s post-test, and *p* values of ≤0.05 were considered statistically significant.

## 3. Results

### 3.1. MNV VPg Is an RNA Binding Protein

**Figure 2:**
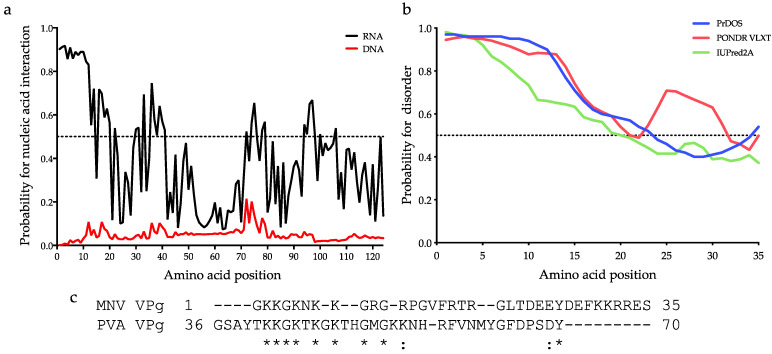
Predictions of nucleic acid binding and disorder of MNV VPg. (**a**) DRNAPred graph depicting predicted RNA (black) and DNA (red) binding probabilities of MNV VPg. (**b**) Predictions for propensity of disorder of the first 35 N-terminal amino acids of MNV VPg using PrDOS (blue), PONDR VLXT (red), and IUPred2A (red) servers. Regions with predictions above 0.5 were considered disordered [33] and are indicated by the dotted line. (**c**) Alignment of the first 35 N-terminal amino acids of MNV VPg and the RNA binding region of PVA VPg. An asterisk (*) indicates fully conserved amino acids and a colon (:) indicates amino acids of a similar nature.

Analysis of the MNV VPg sequence for predicted interactions with RNA and DNA using DRNAPred indicates that MNV VPg has potential for RNA binding, despite the absence of canonical RNA binding motifs such as a zinc finger domain or RNA recognition motif. In particular, the first 12 amino acids have probabilities between 0.91–0.83 for an interaction with RNA, whereas no regions of MNV VPg were predicted to interact with DNA (Figure 2a). The VPg protein of the plant potyvirus, potato A virus (PVA), has been shown to bind RNA [23]. An alignment with the RNA binding motif of PVA VPg identified that the 20 N-terminal amino acids of MNV VPg have some conservation with several basic and glycine residues (Figure 2c), suggesting that this region may have RNA binding potential.

Bioinformatic analysis indicates that MNV VPg contains regions of disorder, consistent with NMR data and disorder predictions of other norovirus VPg proteins [10,40]. The N-terminal 20 amino acids have a disorder tendency of >0.5 and are therefore likely to be intrinsically disordered (Figure 2b). Residues 20–35 in this analysis have low disorder predictions and correspond to the first alpha helix of VPg, as determined by NMR [10]. The combined RNA binding and disorder predictions along with the absence of canonical RNA binding motifs raises the possibility that the N-terminal 20 amino acids of MNV VPg represents a disordered RNA binding element.

As RNA binding by norovirus VPg proteins was not known, we sought to determine the RNA binding potential of MNV VPg and whether it was sequence specific. We used a series of RNA probes, termed pentaprobes [38], which were algorithm-designed to predict every possible five nucleotide long RNA binding motif. These probes, expressed as 12 probes, each 100 nucleotides in length, allowed testing for binding to RNA across a broad range of sequences and to explore specificity of the interaction, as represented by linear RNA sequences.

**Figure 3:**
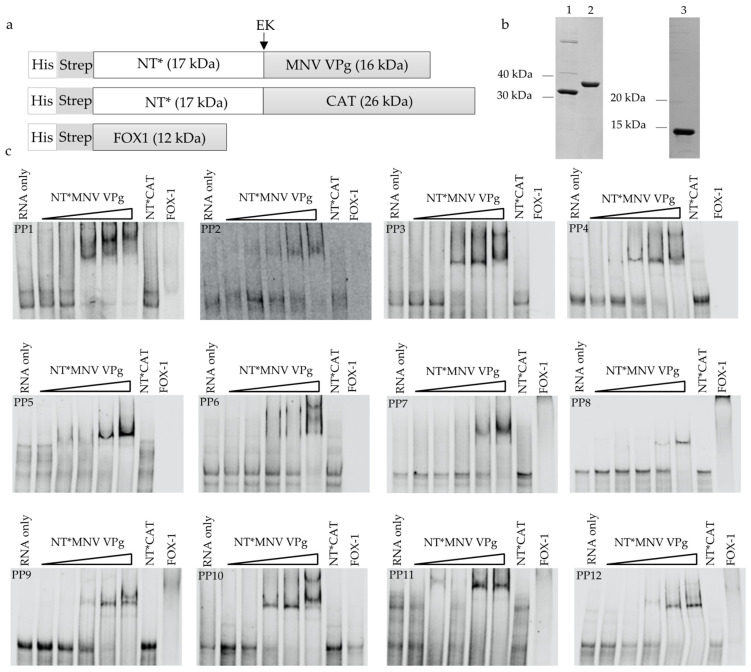
Binding of NT*MNV VPg to RNA pentaprobes. (**a**) Schematic of the NT*MNV VPg, NT*CAT and FOX-1 proteins used in RNA binding experiments. The construct contained a His_6_ and Strep II affinity tag, the NT* fusion partner, and the protein to be expressed. An enterokinase cleavage site (EK) was included for cleavage and removal of the fusion partner. (**b**) Coomassie blue stained SDS-PAGE gel of purified protein (3 μg) used for RNA binding experiments. 1; NT*MNV VPg, 2; NT*CAT, 3; FOX-1. (**c**) RNA EMSA of purified NT*MNV VPg incubated with 100 nM UTP ATTO 680 labelled pentaprobe RNA. The concentration of NT*MNV VPg was increased from 0.5 μM to 8 μM in 2-fold increments and is indicated by a gradient triangle from low concentration to high concentration. NT*CAT (8 μM) and FOX-1 (8 μM) were included as negative and positive controls, respectively.

Recombinant MNV VPg protein was expressed and purified with an N-terminal NT* fusion partner and the resulting protein termed NT*MNV VPg (Figure 3a). The NT* fusion partner is derived from spider silk protein and acts to reduce protein aggregation within the spider silk gland and has proven effective as a fusion partner for proteins expressed in *E. coli* [36]. Production of MNV VPg with the NT* fusion partner generating approximately 40 mg/L of soluble, purified protein illustrates that NT* is an effective fusion partner for baculovirus expression of proteins.

The expression and purity of recombinant proteins used for RNA binding experiments was confirmed by SDS-PAGE gel with Coomassie blue staining (Figure 3b). The interaction of NT*MNV VPg with all 12 pentaprobes was analysed by EMSA, to determine the ability of MNV VPg to bind RNA (Figure 3c). Increasing concentrations of NT*MNV VPg were incubated with 100 nM of pentaprobe RNA and analysed for an upward shift of the RNA, indicative of binding. FOX-1, a known RNA binding protein, previously shown to interact with all 12 pentaprobes [38,41,42], was included as a positive control. FOX-1 induced a mobility shift, or completely abolished RNA migration of the target RNA for all pentaprobes. Upon incubation with NT*MNV VPg, an RNA mobility shift was observed for each pentaprobe (Figure 3c).

In some instances, a second shift band was apparent, which could indicate higher order structures or binding of multiple VPg proteins to a single molecule of RNA. As a negative control protein, NT*CAT showed no RNA binding activity across all 12 pentaprobes when compared with the RNA only control, confirming that a protein-RNA binding event was not a consequence of the NT* fusion tag. Removal of NT* using enterokinase resulted in MNV VPg still binding to RNA, confirming that NT* did not cause the binding of VPg to pentaprobes (Figure S1). Cleavage of VPg from the NT* tag with enterokinase showed multiple non-specific cleavage sites (Figure S1). Given that RNA binding still occurs with the N-terminal fusion, future experiments were undertaken with NT*MNV VPg. Overall, the RNA EMSA assays show that MNV VPg is a non-specific RNA binding protein. As the protein was bound to all pentaprobe RNA targets, a subgroup of pentaprobes (PP4, PP7 and PP9) were used for subsequent analyses.

**Figure 4:**
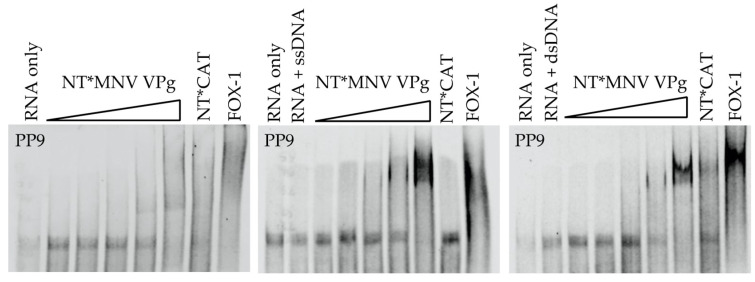
NT*MNV VPg preferentially binds RNA. NT*MNV VPg was incubated with 100 nM UTP ATTO 680 labelled pentaprobe RNA and a 100-fold molar excess of either single-stranded or double-stranded unlabelled DNA probe for 30 min. The concentration of NT*MNV VPg was increased from 0.5 μM to 8 μM in 2-fold increments, as shown by a gradient triangle. NT*CAT (8 μM) and FOX-1 (8 μM) were included as negative and positive controls, respectively.

Predictive analyses of VPg indicated a low probability for DNA binding (Figure 2a). The specificity for RNA over DNA was investigated by a competition EMSA assay. To achieve this, unlabelled single-stranded or double-stranded DNA was added to the reaction at a 100-fold molar excess over labelled PP9 RNA. An RNA mobility shift was observed when NT*MNV VPg was incubated with the labelled PP9 RNA pentaprobe and either ssDNA or dsDNA (Figure 4). Despite a molar excess of DNA, NT*MNV VPg still bound to RNA, indicating a strong preference for RNA and no evidence for DNA binding in this competition assay.

**Figure 5:**
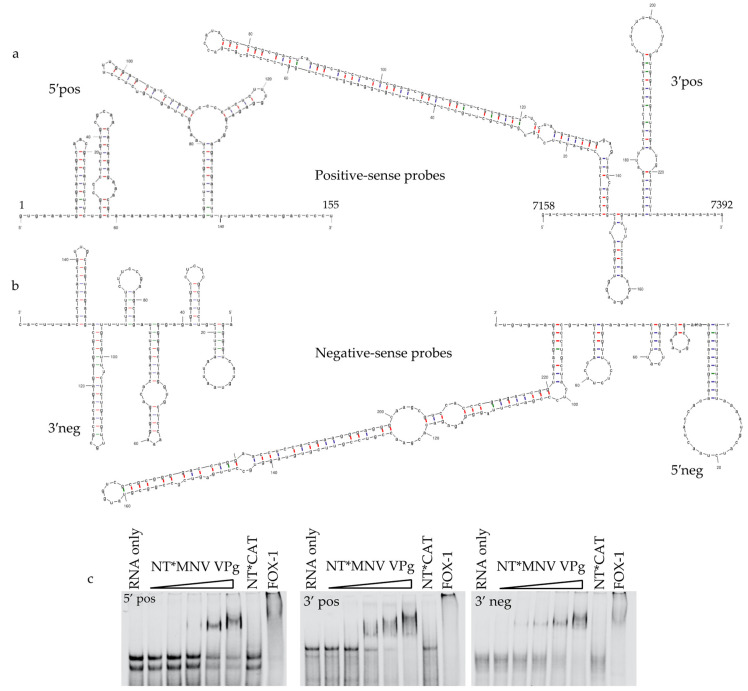
Viral RNA probes. (**a**) Schematic of the positive-sense viral RNA probes (5′pos and 3′pos). The genome positions of nucleotides for the positive-sense RNA probes are indicated. (**b**) Schematic of the negative-sense viral RNA probes 3′neg and 5′neg. The negative-sense RNA probes are the complements of the positive-sense RNA probes. RNA secondary structures (**a**,**b**) were predicted using mfold [44,45]. (**c**) NT*MNV VPg was incubated with labelled 5′pos, 3′pos or 3′neg RNA probes and analysed by EMSA. The concentration of NT*MNV VPg was increased from 0.5 μM to 8 μM in 2-fold increments. NT*CAT (8 μM) and FOX-1 (8 μM) were included as negative and positive controls, respectively.

VPg is essential for replication of the viral RNA and is covalently linked to the 5′ end of the viral RNA genome [13,43]. RNA probes were designed to represent the 5′ or 3′ termini of the viral positive-sense or negative-sense RNA, to determine if VPg could interact with these genomic ends (Figure 5). These probes were designed to incorporate the 5′ and 3′ UTRs of the genomic RNA along with several major stem-loop structures. Labelled RNA probes were synthesized, purified, and the interaction of NT*MNV VPg with the 5′pos, 3′pos, and 3′neg probes was examined (Figure 5c). By EMSA NT*MNV VPg bound to each of these RNA probes. We were unable to produce RNA for the 5′neg probe, corresponding to the 5′ end of the negative-sense RNA. This confirms that VPg can bind to the terminal regions of the genomic and antigenomic RNA; however, there is no evidence that this is specific or enhanced in comparison to the binding affinity shown for pentaprobe RNA.

**Figure 6:**
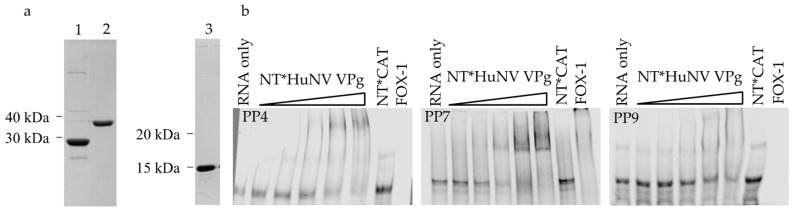
HuNV VPg binds RNA. (**a**) Coomassie blue stained SDS-PAGE gel of purified protein (3 μg) used for RNA binding experiments. 1; NT*HuNV VPg, 2; NT*CAT, 3; FOX-1. (**b**) RNA EMSA of NT*HuNV VPg with pentaprobe RNA. Proteins were incubated with 100 nM UTP ATTO 680 labelled pentaprobe RNA for 30 min, separated on a 5% TBE gel, and directly imaged in the 700 nm channel. The concentration of NT*HuNV VPg was increased from 0.5 μM to 8 μM in 2-fold increments. NT*CAT (8 μM) was included as a negative control and FOX- 1 (8 μM) as a positive control.

Due to the conservation in sequence and common functions shown for norovirus VPg proteins, we tested if RNA binding would be a conserved function of other norovirus VPg proteins. A recombinant baculovirus was generated to express GII.4 Sydney 2012 strain HuNV VPg with an N-terminal NT*fusion protein. Purity of NT*HuNV VPg was confirmed by SDS-PAGE gel (Figure 6a) and the interaction with PP4, PP7, and PP9 RNA was investigated by EMSA (Figure 6b). As with previous experiments, there was no shift in the unbound RNA band upon incubation with NT*CAT and a complete disappearance with FOX-1. Addition of NT*HuNV VPg showed a gel shift with PP4, PP7, and PP9 RNA. The shift was identified by a decrease in the unbound RNA band and, for PP4 and PP7, the appearance of a shifted band visible when RNA was incubated with NT*HuNV VPg. Similar to MNV VPg, the binding of HuNV VPg to multiple probes is interpreted as binding to RNA in a non-specific manner.

### 3.2. The Basic Amino Acid Patch Near the N-Terminus of MNV VPg Is Required for Binding to RNA

**Figure 7:**
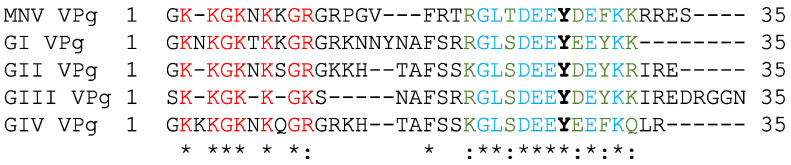
Alignment of VPg sequences. An asterisk (*) indicates identical residues and a colon (:) indicates residues that are similar. Conserved amino acids of the N-terminal region are shown in red. The tyrosine residue (Y) corresponding to Y26 of MNV VPg is shown in black in bold. The fully conserved amino acids surrounding the Y26 are shown in blue and similar amino acids in green. The represented genogroups are GI Norwalk virus VPg (AAC64602), GII Sydney 2012 VPg (JX459908), GIII Jena virus VPg (CAA90480) and GIV Lake Macquarie VPg (AFJ21375).

The N-terminus of norovirus VPg contains a highly conserved epitope rich in positively charged lysine and arginine residues (Figure 7). Predictive analyses indicate that the disordered N-terminus of MNV VPg containing the conserved epitope may be responsible for binding to RNA (Figure 1 and Figure 7).

**Figure 8:**
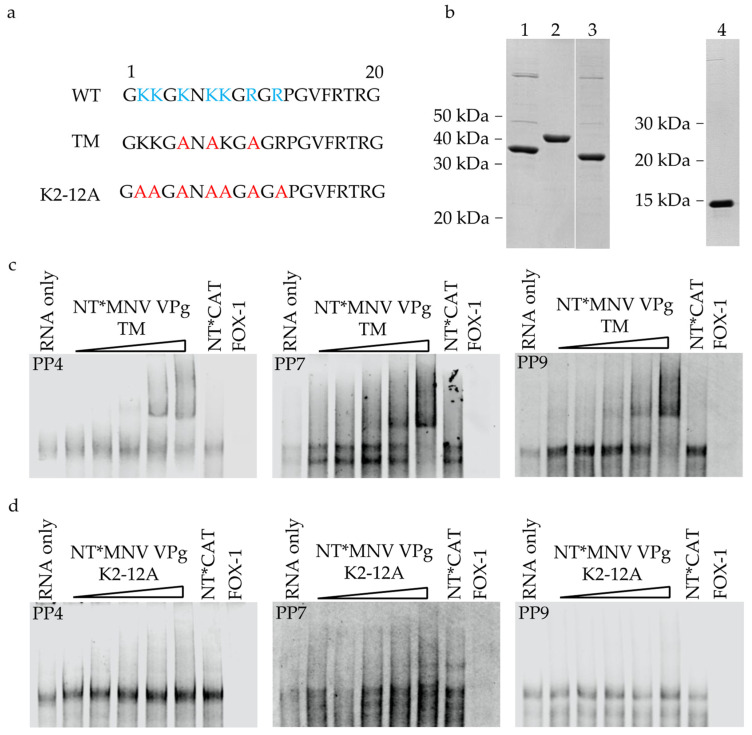
N-terminal lysine and arginine amino acids are important for binding to RNA. (**a**) Schematic of the first 20 amino acids of MNV VPg, wild-type amino acids are shown in blue. Amino acids mutated to alanine are shown in red for the NT*MNV VPg and NT*MNV VPg K2-12 constructs, respectively. (**b**) Coomassie blue stained SDS-PAGE gel of purified protein (3 μg) used for RNA binding experiments. 1; NT*MNV VPg TM; NT*CAT, 3; NT* MNV VPg K2-12 and 4; FOX-1. (**c**,**d**) RNA EMSA of NT*MNV VPg K2-12 and NT*MNV VPg TM with pentaprobe RNA. Proteins were incubated with 100 nM UTP ATTO 680 labelled pentaprobe RNA for 30 min, separated on a 5% TBE gel and directly imaged in the 700 nm channel. The concentration of NT*MNV VPg TM was increased from 0.5 μM to 8 μM in 2-fold increments. NT*CAT (8 μM) was included as a negative control and FOX-1 (8 μM) as a positive control.

Two MNV VPg constructs were designed to test the importance of positively charged amino acids within the conserved sequence region for binding to RNA. The first construct created mutations, to alanine, at residues K5, K7, and R10 of the conserved epitope of MNV VPg (MNV VPg TM) (Figure 8a). The second construct generated alanine mutations at all lysine and arginine residues within the first 12 amino acids of MNV VPg (MNV VPg K2-12). Proteins were expressed with an N-terminal NT* fusion protein and purification of NT*MNV VPg TM and NT*MNV VPg K2-12 was confirmed by SDS-PAGE gel (Figure 8b). The interactions with PP4, PP7, and PP9 were analysed by EMSA (Figure 8c,d). Incubation of NT*MNV VPg TM with RNA resulted in a gel shift for all three pentaprobes. This indicates that MNV VPg TM is still capable of binding to RNA and mutation of just three amino acids did not eliminate RNA binding. In contrast, addition of NT*MNV VPg K2-12 had no effect on the mobility of pentaprobe RNA at the protein concentrations tested. This suggests that the seven lysine and arginine residues within the conserved N-terminal region of MNV VPg may form a basic patch and act co-operatively to facilitate non-specific RNA binding.

**Figure 9:**
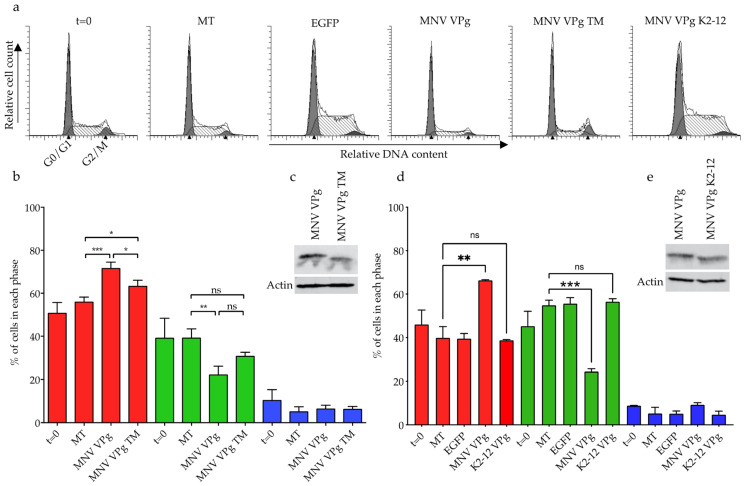
Manipulation of the cell cycle by MNV VPg with N-terminal mutations. RAW-Blue cells were transfected with 4–5 μg of transcript RNA corresponding to MNV VPg, MNV VPg TM, or MNV VPg K2-12. Mock transfected (MT) cells were seeded at the time of transfection as a negative control. At 12 h post transfection, cells were harvested for western blot and flow cytometry analysis of the cell cycle. (**a**) Representative histograms from one of three experiments. The positions of the G0/G1 and G2/M phase populations of cells are labelled on the *t* = 0 histogram. The S phase lies between the G0/G1 and G2/M populations and is indicated by hatched lines. (**c**,**e**) Analysis of total cell lysate by western blot to confirm expression of MNV VPg, MNV VPg TM, and MNV VPg K2-12, with actin as a loading control. (**b**,**d**) The histograms were analysed using MODfit LT 3.0 and the percentage of cells in each phase of the cell cycle are shown. Red indicates percentage of cells in G0/G1 phase, green indicates the S phase, and blue indicates the G2/M phase. The results present the mean and SD from three independent experiments. Statistical significance was determined using a one-way ANOVA with Dunnett’s post-test. ns; not significant, * *p* ≤ 0.05, ** *p* ≤ 0.01 and *** *p* ≤ 0.001.

The conserved N-terminal region of norovirus VPg proteins is involved in binding of NTPs, nucleotidylation, and cell cycle manipulation [11,17,25]. We were interested in determining if mutations that eliminate RNA binding have any effect on the cell cycle, as previously this has only been investigated in relation to single point mutations or truncations of the motif [17]. To determine the importance of positively charged amino acids for a G0/G1 cell cycle arrest, mRNA transcripts encoding MNV VPg TM and MNV VPg K2-12 were transfected into asynchronous RAW-Blue cells and the percentage of cells in each phase of the cell cycle measured by flow cytometry (Figure 9). Expression of VPg constructs was confirmed by western blot and each construct was shown to express to similar levels as wild-type MNV VPg (Figure 9c,e).

Cell cycle analysis showed that at 12 h post-transfection, MNV VPg induced an increase, to approximately 70% of cells in G0/G1 phase with a corresponding decrease in S phase cells. (Figure 9b,d). MNV VPg TM induced an accumulation of cells in G0/G1, to 63%, significantly different from both mock transfected cells and MNV VPg, indicating that while there was still a change in the G0/G1 population it was decreased (Figure 9b). In contrast, transfection of MNV VPg K2-12 showed no significant changes in the percentage of cells in each phase of the cell cycle compared to mock transfected cells (Figure 9d). That MNV VPg TM binds RNA and induces a partial cell cycle arrest while MNV VPg K2-12 does not possess either of these functions corroborates previous findings on the importance of the N-terminal region for cell cycle arrest and that this correlates with the basic patch. However, further research will need to be performed to confirm whether there is a direct mechanistic relationship between RNA binding and cell cycle manipulation.

## 4. Discussion

### 4.1. MNV VPg Is an RNA Binding Protein

We used EMSA gels to show that MNV VPg binds to RNA as predicted by bioinformatic analysis and consistent with observed phosphocellulose binding of this protein [20].

The VPg proteins used in this study were expressed as a fusion with the NT* protein. Here we show that the addition of the NT* fusion protein to norovirus VPg enabled good expression in *T.ni* cells. MNV VPg was capable of binding to RNA both with the NT* fusion or without (Figure 3 and Figure S1), indicating that the presence of the fusion did not affect this specific function.

The binding of NT*MNV VPg was specific for RNA even with an excess of DNA in the experiment, suggesting that RNA is the favoured binding partner. RNA binding occurred in a non-specific manner, with binding of MNV VPg to all 12 pentaprobe sequences tested, representing all possible five nucleotide combinations, the most common length of RNA sequences targeted by RNA binding proteins. This result is consistent with the VPg proteins from the *Potyviridae* and *Picornaviridae* viral families that also bind RNA in a non-specific manner [22,23,46]. We have also shown that this RNA binding is not unique to MNV with the VPg protein of the GII.4 Sydney 2012 norovirus also showing a non-specific interaction with RNA.

A wide range of proteins show non-specific RNA binding, with an estimated half of RNA binding proteins falling into this category [47]. Examples of non-specific RNA binding protein include translation initiation factors such as eIF4E [48] and proteins involved in RNA splicing, including the SR protein family [26,49,50]. Non-specific RNA binding proteins typically interact with sites that appear to be devoid of specific sequence or structural motifs. However, the non-specific interaction of VPg with RNA pentaprobes does not preclude a specific target(s) for VPg. There are multiple ways in which specific RNA binding can be achieved, including the specific recognition of RNA secondary structure. For example, the FUS protein binds specifically to AU-rich stem-loops but without a specific sequence motif [51]. The ends of the norovirus genome contain several evolutionarily conserved RNA structures [52,53]. We were unable to identify any enhanced binding to RNA probes corresponding to the 5′ and 3′ ends of the viral genome using the assays employed in this study.

RNA binding proteins can also have specificity conferred by interaction with other proteins. The VPg protein from poliovirus shows specific RNA binding activity in conjunction with the viral polymerase for RNA genome structures [54,55,56]. The MNV polymerase protein has RNA binding activity [57] and there is interaction between the VPg protein and the RNA polymerase for nucleotidylylation of the VPg and for initiation of RNA synthesis [10,11,58], and it is possible that this interaction confers specificity upon the VPg interaction.

### 4.2. Identification of a Motif/Region of MNV VPg Required for Binding to RNA

The N-terminus of MNV VPg was predicted to interact with RNA but does not contain any sequences traditionally associated with RNA binding motifs. Instead, there is a correlation between the potential for RNA binding and propensity for disorder by the N-terminal amino acids (Figure 2). Many RNA binding proteins contain regions of intrinsic disorder [59]. One of these non-canonical RNA binding regions has been termed the K/R basic patch. Basic patches are normally composed of 4–8 lysine amino acids and occasionally arginine amino acids, which form a highly positive and exposed surface for binding to RNA [26,60]. The conserved N-terminal region of MNV VPg contains five lysine and two arginine residues within the first 12 amino acids, consistent with a disordered basic patch (Figure 7). Given the resemblance to a K/R basic patch, it seemed likely that the N-terminal region of MNV VPg, which is enriched in lysine and arginine amino acids would be required for RNA binding activity. In support of this prediction, PVA VPg has been shown to bind RNA in a sequence independent manner, which is dependent on a stretch of amino acids with a high local positive charge [23,46]. Alanine substitutions to MNV VPg at positions K5, K7, and R10 did not inhibit RNA binding while mutation of all lysine and arginine residues within the first 12 amino acids inhibited binding consistent with non-canonical RNA binding. These results indicate that K5, K7, and R10 residues are not, by themselves, essential for RNA binding, and the interaction appears to involve a larger region of the N-terminus of VPg. Future work should look to identify the minimum requirements for charged residues to enable RNA binding by MNV VPg.

In Jembrana disease virus, RNA binding is mediated by arginine residues, which form electrostatic interactions with RNA that is stabilized by the surrounding amino acids [61]. Similarly, with PVA VPg, it has been proposed that the protein has a loosely folded and positively charged contact surface at the edge of the protein [23]. Mutation of lysine residues in this area is predicted to drop the surface charge of PVA VPg, resulting in decreased attraction between the VPg and either RNA or individual NTPs [23]. The conserved N-terminal motif of MNV VPg shares similarities with the RNA binding motif of PVA VPg, particularly the lysine residues (Figure 2). Given the decreased RNA binding when charged amino acids are replaced with alanine, it seems likely that the N- terminal residues of MNV VPg allow binding to RNA in a similar manner via electrostatic interactions.

### 4.3. Biological Significance of RNA Binding by MNV VPg

RNA binding appears to be a conserved function of norovirus, potyvirus, and picornavirus VPg proteins. The biological significance of RNA binding by norovirus and potyvirus VPg proteins is not clear. The charged residues in the N-terminal region of MNV VPg have been linked with nucleotidylylation, NTP binding, and induction of a cell cycle arrest. We have investigated a possible link with induction of a G0/G1 phase cell cycle arrest. Mutation of all K and R residues in the first 12 amino acids of MNV VPg results in no cell cycle arrest and no RNA binding. The MNV VPg TM with only three charged amino acids replaced, induced a partial cell cycle arrest and still bound to RNA. Future experiments should address the affinity for RNA binding by mutant and wild type VPg proteins to further elucidate a potential relationship between these functions.

RNA binding proteins (RBPs) play a crucial role in the regulation of cell cycle progression [62]. These proteins participate in a variety of post-transcriptional RNA processing steps, including translation, RNA stability, localisation, and splicing for both mRNA and non-coding RNAs. This study has shown that the N-terminal K/R basic patch of MNV VPg is important for induction of a cell cycle arrest and binding to RNA; perturbation of the patch affects both of these biological activities. However, a causal relationship between RNA binding and the other functions associated with the K/R patch is yet to be established. The sequence conservation in noroviruses and the ability of VPg proteins from a range of RNA viruses to bind RNA non-specifically points towards an important role for VPg RNA binding in the replication of these RNA viruses.

## Figures and Tables

**Table 1:** Primer sequences for generation of RNA probes corresponding to the viral genome.

	Forward Primer Sequence (5′–3′) ^a^	Reverse Primer Sequence (5′–3′) ^b^
5′pos	GAATTCTAATACGACTCACTATAGTGAAA- TGAGGATGGCAACG	GGATCC**GATATC**AGGGGTCATGTAATTAAT- TTCGTC
3′pos	GAATTCTAATACGACTCACTATAGACACA- TCCCCTCTACCGA	GGATCC**GATATC**TTTTTTTTTTAAAATGCAT- CTAACT
5′neg	GAATTCTAATACGACTCACTATAGTTTTTTT- TTTAAAATGCATCTAACTACCAC	GGATCC**GATATC**GACACATCCCCTCTACCG- ATCTC
3′neg	GAATTCTAATACGACTCACTATAGAGGGG- TCATGTAATTAATTTCGTC	GGATCC**GATATC**GTGAAATGAGGATGGCA- ACG

^a^ T7 promoter sequence is underlined; ^b^ EcoRV restriction site is shown in bold.

## Data Availability

The data presented in this study are available in the manuscript and Supplementary Figure S1.

## Supplementary Materials

The following are available online at https://www.mdpi.com/article/10.3390/v13071282/s1, Figure S1, MNV VPg binds RNA.
