# Supplementary material for: Norovirus VPg Binds RNA through a Conserved N-Terminal K/R Basic Patch
Source: Viruses. 2021 Jun 30;13(7):1282. doi: 10.3390/v13071282 (PMC8310136; doi:10.3390/v13071282)
Supplement: Supplementary file 1 [file viruses-13-01282-s001.zip › viruses-1272009-supplementary.pdf]

viruses

Supplementary Materials: Norovirus VPg binds RNA through a conserved N-terminal K/R basic patch

Supplementary Materials:

NT* solubility tag removal: The protein was buffer exchanged into enterokinase cleavage buffer (20 mM Tris pH 8, 50 mM NaCl, 2mM $CaCl_2$) and cleaved using bovine enterokinase (New England Biolabs, Ipswich, MA, USA) at 16 U/mg protein for 4 hours. The enterokinase was removed using soybean trypsin inhibitor agarose (Sigma, St Louis, MO, USA) and the cleaved NT* tag was removed using Ni-NTA resin. The purified VPg proteins were then buffer exchanged into 50mM Tris, 150 mM NaCl and 10% glycerol and stored at −80 °C.

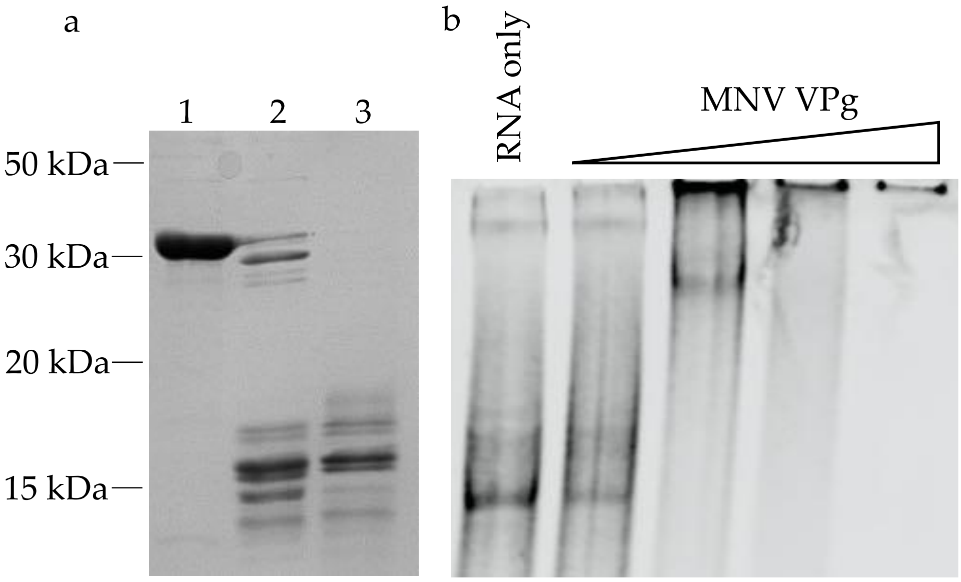

Figure S1. MNV VPg binds RNA. (**a**) NT*MNV VPg was digested with bovine enterokinase to remove the N-terminal fusion tags and analysed by SDS-PAGE gel followed by staining of proteins with Coomassie blue. 1; no enterokinase added to the protein, 2; enterokinase cleavage reaction, 3; purified MNV VPg sample. (**b**) MNV VPg was incubated with 100 nM of ATTO 680 labelled PP1 RNA for 30 min, separated on a 5% TBE gel and directly imaged in the 700 nm channel. MNV VPg was added to reactions at increasing concentrations; 2 µM, 5 µM, 10 µM and 20 µM.
